# Newborn screening for hearing and sight, Cameroon

**DOI:** 10.2471/BLT.24.292431

**Published:** 2025-05-03

**Authors:** Gaelle Vofo, Brice Vofo, Winnie Anoumedem, Holgar Fung, Demosthene Afofou, Christabel Abanda, Serge Fankeng, Winnie Mbwentchou, Eugenie Mempouo, Sara Kingue, Yanelle Wandji, Pamela Ngounou, Maurice Mpessa, Wilfried Ganni, Francine Mveng, Evelyne Nguedia, Caren Mason, Evariste Nguimkeu, Frenkel Shahar, Sagit Stern, Michal Kaufmann, Clement Assob, Christian Andjock, Menachem Gross, Richard Njock

**Affiliations:** a Shomea, Marché Mfoundi, Yaoundé, Cameroon.; b Direction of Development of Sub-Saharan African Hearing rehabilitation solutions, Oticon, Paris, France.; c Department of Otolaryngology-Head & Neck Surgery, Yaoundé General Hospital, Yaoundé, Cameroon.; d Faculty of Health Sciences, University of Buea, Buea, Cameroon.; e Department of Otolaryngology-Head & Neck Surgery, Bonassama District Hospital, Bonabéri, Cameroon.; f Department of Otolaryngology-Head & Neck Surgery, Laquintinie Hospital, Douala, Cameroon.; g Department of Otolaryngology-Head & Neck Surgery, Djoungolo Hospital, Yaoundé, Cameroon.; h Department of Ophthalmology, Hadassah Medical Center, Jerusalem, Israel.; i Department of Otolaryngology, Head & Neck Surgery, Hadassah Medical Center, Jerusalem, Israel.; j Ministry of Public Health, Yaoundé, Cameroon.

## Abstract

**Objective:**

To evaluate a combined hearing and eye screening model for newborns attending immunization clinics in Cameroon.

**Methods:**

We analysed data from a screening project that took place between November 2021 and February 2024, which assessed both the hearing and eyes of newborns using otoacoustic emission and fundal reflex tests, respectively. We then evaluated sensitivity, specificity and predictive values of screening conducted by trained auxiliary staff versus specialists.

**Findings:**

We screened 1807 newborns, of which 54% (976) were female. The median age at screening was 13 days. Eight percent of newborns (141/1807) did not pass the otoacoustic emission test; screeners scheduled these newborns for a second-line otoacoustic emission test within three months. Only 28% (39/141) returned for the repeat otoacoustic emission test. Of the returning babies, 33% (13/39) still did not pass, and screeners referred them for an auditory brainstem response threshold test. Screeners detected an absent fundal reflex in 2% (27) of babies. Compared to specialists, trained auxiliary staff showed 82% sensitivity and 99% specificity in hearing screening; predictive values were 90% (positive) and 99% (negative). For eye screening, sensitivity was 67% and specificity 99%, with predictive values of 86% (positive) and 98% (negative).

**Conclusion:**

Combined screening performed by trained auxiliary staff in immunization clinics offers a promising approach to screening newborns’ hearing and eyes, enabling broader population coverage with fewer resources. Combined screening conducted at immunization clinics includes both hospital- and community-born babies and is therefore suitable for countries with a high number of out-of-hospital births.

## Résumé

**Objectif:**

Évaluer un modèle de dépistage combiné des troubles auditifs et oculaires pour les nouveau-nés en consultation dans les cliniques de vaccination au Cameroun.

**Méthodes:**

Nous avons analysé les données d’un projet de dépistage qui s’est déroulé entre novembre 2021 et février 2024. Il évaluait à la fois l’audition et la vue des nouveau-nés en mesurant les émissions oto-acoustiques et en testant le réflexe du fond de l’œil, respectivement. Nous avons ensuite évalué la sensibilité, la spécificité et les valeurs prédictives du dépistage effectué par du personnel auxiliaire formé, par rapport à des résultats obtenus par des spécialistes.

**Résultats:**

Nous avons dépisté 1807 nouveau-nés, dont 54% (976) étaient de sexe féminin. L’âge médian au moment du dépistage était de 13 jours. Huit pour cent des nouveau-nés (141/1807) n’ont pas réussi le test d’émissions oto-acoustiques; les dépisteurs ont inscrit ces nouveau-nés à un tel test de deuxième intention dans les trois mois. Seuls 28% (39/141) d’entre eux sont revenus pour repasser le test d’émissions oto-acoustiques. Parmi les bébés revenus, 33% (13/39) ne l’ont toujours pas réussi, et les dépisteurs les ont orientés vers un test de seuil de réponse auditive du tronc cérébral. Ils ont détecté une absence de réflexe du fond de l’œil chez 2% (27) des bébés. Par rapport aux spécialistes, le personnel auxiliaire formé a fait preuve d’une sensibilité de 82% et d’une spécificité de 99% dans le dépistage auditif; les valeurs prédictives étaient de 90% (positives) et de 99% (négatives). Pour le dépistage oculaire, la sensibilité était de 67% et la spécificité de 99%, avec des valeurs prédictives de 86% (positives) et 98% (négatives).

**Conclusion:**

Le dépistage combiné effectué par du personnel auxiliaire formé dans les cliniques de vaccination constitue une approche prometteuse pour le dépistage des troubles auditifs et oculaires chez les nouveau-nés, permettant une couverture plus large de la population en nécessitant moins de ressources. Le dépistage combiné effectué dans les cliniques de vaccination concerne à la fois les bébés nés à l’hôpital et en-dehors et convient donc aux pays où le nombre de naissances hors hôpital est élevé.

## Resumen

**Objetivo:**

Evaluar un modelo combinado de cribado auditivo y ocular para recién nacidos que asisten a consultas de vacunación en Camerún.

**Métodos:**

Se analizaron los datos de un proyecto de cribado realizado entre noviembre de 2021 y febrero de 2024, que evaluó tanto la audición como los ojos de los recién nacidos mediante pruebas de emisiones otoacústicas y de reflejo fundoscópico, respectivamente. Posteriormente, se evaluaron la sensibilidad, especificidad y valores predictivos del cribado realizado por personal auxiliar capacitado en comparación con especialistas.

**Resultados:**

Se cribaron 1807 recién nacidos, de los cuales el 54% (976) eran niñas. La edad mediana al momento del cribado fue de 13 días. El 8% de los recién nacidos (141/1807) no superó la prueba de emisiones otoacústicas; el personal de cribado programó para estos recién nacidos una segunda prueba de emisiones otoacústicas dentro de los tres meses siguientes. Solo el 28% (39/141) acudió a la repetición de la prueba. De los recién nacidos que regresaron, el 33% (13/39) volvió a no superar la prueba y fueron derivados para una prueba de umbral de respuesta auditiva del tronco encefálico. El personal de cribado detectó ausencia de reflejo fundoscópico en el 2% (27) de los recién nacidos. En comparación con los especialistas, el personal auxiliar capacitado mostró una sensibilidad del 82% y una especificidad del 99% en el cribado auditivo; los valores predictivos fueron del 90% (positivo) y del 99% (negativo). Para el cribado ocular, la sensibilidad fue del 67% y la especificidad del 99%, con valores predictivos del 86% (positivo) y del 98% (negativo).

**Conclusión:**

El cribado combinado realizado por personal auxiliar capacitado en consultas de vacunación representa un enfoque prometedor para el cribado de la audición y la visión de los recién nacidos, permitiendo una mayor cobertura poblacional con menos recursos. El cribado combinado realizado en las consultas de vacunación incluye tanto a recién nacidos hospitalarios como nacidos en la comunidad y resulta, por tanto, adecuado para países con un elevado número de nacimientos extrahospitalarios.

## ملخص

الغرض تقييم نموذج مُدمج لفحص السمع والعين لحديثي الولادة الذين يرتادون عيادات التحصين في الكاميرون.

الطريقة قمنا بتحليل البيانات من مشروع للفحص تم تنفيذه بين نوفمبر/تشرين ثاني 2021 وفبراير/شباط 2024، والذي قام بتقييم سمع وعين حديثي الولادة باستخدام اختباري الانبعاث السمعي الأذني وانعكاس قاع العين، على التوالي. ثم قمنا بتقييم حساسية وخصوصية وقيم الفحص التي أجراها موظفون مساعدون مُدرَّبون مقارنةً بالفحص الذي أجراه متخصصون.

النتائج قمنا بفحص 1807 من حديثي الولادة، %54 منهم (976) من الإناث. كان متوسط عمر المواليد عند الفحص 13 يومًا. لم ينجح %8 من حديثي الولادة (141/1807) في اجتياز اختبار الانبعاث السمعي الأذني؛ لذا حدد القائمون على الفحص موعدًا لهؤلاء المواليد لاختبار الانبعاث السمعي الأذني من الخط الثاني في غضون ثلاثة أشهر. عاد %28 فقط (39/141) لإجراء اختبار الانبعاث السمعي المتكرر. ومن بين الأطفال العائدين، لم ينجح %33 (13/39) بعد، وأحالهم القائمون بالفحص لإجراء اختبار عتبة استجابة جذع الدماغ السمعية. واكتشف القائمون بالفحص غياب رد الفعل القاعي لدى %2 (27) من الأطفال. وبالمقارنة مع المتخصصين، أظهر فريق العمل من المساعدين المدربين حساسية بنسبة %82، ودقة بنسبة %99 في فحص السمع؛ وكانت القيم التنبؤية %90 (إيجابية) و%99 (سلبية). أما بالنسبة لفحص العين، فقد كانت الحساسية %67 والدقة %99، مع قيم تنبؤية %86 (إيجابية) و%98 (سلبية).

الاستنتاج إن الفحص المشترك الذي يجريه فريق عمل من المساعدين المدربين في عيادات التحصين، يوفر نهجًا واعدًا لفحص سمع وعيون الأطفال حديثي الولادة، مما يتيح تغطية سكانية أوسع بموارد أقل. إن الفحص المشترك الذي يتم إجراؤه في عيادات التحصين يشمل الأطفال المولودين في المستشفى وفي المجتمع، وبالتالي فهو مناسب للدول التي بها عدد كبير من الولادات خارج المستشفى.

## 摘要

**目的:**

为了评估喀麦隆新生儿免疫接种门诊听力与视力联合筛查模式的效果。

**方法:**

在 2021 年 11 月至 2024 年 2 月期间开展的一项筛查项目分别运用耳声发射和眼底反射检测方法对新生儿的听力和视力进行了评估，我们分析了该项目的相关数据。然后我们对由受过培训的辅助工作人员与专家开展的筛查工作的灵敏度、特异度和预测值进行了评估。

**结果:**

我们对 1,807 名新生儿进行了筛查，其中女婴所占比例为 54% (976)。接受筛查的新生儿中位年龄为 13 天。百分之八的新生儿 (141/1,807) 没有通过耳声发射检测；筛查人员预定在三个月内为这些新生儿安排一次二线耳声发射检测。只有 28% (39/141) 的新生儿如约再次接受了耳声发射检测。在再次接受检测的婴儿中，仍有 33% (13/39) 的婴儿没有通过检测，筛查人员推荐这些婴儿接受听觉脑干反应阈值检测。筛查人员发现，2% (27) 的婴儿存在眼底反射消失的现象。与专家相比，受过培训的辅助工作人员在听力筛查中表现出的灵敏度为 82%，特异度为 99%；预测值则为 90%（阳性）和 99%（阴性）。至于视力筛查，灵敏度为 67%，特异度为 99%，预测值则为 86%（阳性）和 98%（阴性）。

**结论:**

由受过培训的辅助工作人员在免疫接种门诊所开展的新生儿听力和视力联合筛查是一种很有前景的筛查方法，能在资源比较稀缺的情况下让更广大的群体都能接受筛查。在免疫接种门诊所开展的联合筛查涵盖了医院出生和社区出生的所有婴儿，因此该方法适用于院外出生率较高的国家。

## Резюме

**Цель:**

Оценить модель комбинированной проверки зрения и слуха у новорожденных, посещающих прививочные кабинеты в Камеруне.

**Методы:**

Авторы проанализировали данные скринингового проекта, который осуществлялся в период с ноября 2021 г. по февраль 2024 г., в котором у новорожденных оценивались одновременно слух и зрение при помощи отоакустической эмиссии и рефлекса глазного дна соответственно. Была проведена оценка чувствительности, специфичности и прогностического значения скрининга, который проводился силами обученного вспомогательного персонала, по сравнению с таковыми значениями при проведении обследования специалистами**.**

**Результаты:**

Было обследовано 1807 новорожденных, из которых 54% (976) были женского пола. Медианный возраст на момент скрининга составлял 13 дней. Восемь процентов новорожденных (141 из 1807) не прошли тест с использованием отоакустической эмиссии; для них был запланирован отоакустический тест второй линии в течение трех месяцев. Из них только 28% (39 из 141) вернулись для повторного отоакустического эмиссионного тестирования. Из тех детей, что вернулись, 33% (13 из 39) снова не прошли тест, и исследователи направили их на проверку порога слуховой реакции стволового отдела мозга. Отсутствие рефлекса глазного дна было выявлено у 2% (27) детей. По сравнению со специалистами обученные вспомогательные медработники показали 82%-ю чувствительность и 99%-ю специфичность в исследованиях слуха; прогностическое значение составило 90% (положительный результат) и 99% (отрицательный результат). При обследовании глаз чувствительность составила 67%, специфичность – 99%, прогностические значения – 86% (положительный результат) и 98% (отрицательный результат).

**Вывод:**

Комбинированное обследование, проводимое обученными вспомогательными медработниками в прививочном кабинете, является многообещающим подходом в обследовании слуха и зрения новорожденных, позволяющим охватить больше населения при меньшем количестве ресурсов. Комбинированное обследование в прививочных кабинетах охватывает детей, рожденных как в больничных, так и в домашних условиях, поэтому пригодно для стран, в которых значительное число детей рождается во внебольничных условиях.

## Introduction

Congenital hearing and visual impairments are among the most prevalent of sensory impairments.^1^^,^^2^ Congenital hearing loss affects 1.1–1.7 per 1000 live births in high-income countries^3^^–^^5^ and 2.0–6.0 per 1000 in low- and middle-income countries.^6^^,^^7^ While public health measures, such as vaccination and vitamin A supplements, have reduced blindness, cataract remains a considerable health burden worldwide.^8^ In high-income countries, congenital cataracts occur in 1.7 per 10 000 births, while retinoblastoma affects 6.0–7.1 per 100 000 births.^9^^–^^11^ High rates of congenital infections in low- and middle-income countries, such as toxoplasmosis, rubella, cytomegalovirus and herpes simplex, account for higher prevalence of congenital cataracts compared with high-income countries (although, often, no cause can be found).^12^

The invisible nature of congenital hearing and visual impairments means that they require screening for early detection and intervention. Early identification of these conditions and their management is vital to prevent poor development of the affected child’s language, social, motor and cognitive skills.

The World Health Organization (WHO),^13^ United States Centers for Disease Control and Prevention^14^ and the American Academy of Pediatrics’ Joint Committee on Infant Hearing^15^ recommend that newborn hearing and eye screening occur within the first four weeks of life. While almost all newborns in the United States of America are routinely screened at birth,^3^ in many low- and middle-income countries, routine hearing and eye screening is not done at birth due to lack of an effective routine screening model. For example, the effectiveness of conventional hospital-based universal hearing screening programmes is questionable in these settings because of the high proportion of non-hospital (community) births^16^ and the high cost of equipment.^17^ Furthermore, the considerable effort and resources needed to train and monitor separate hearing and eye screening personnel, and the high risk of equipment misappropriation in busy settings, pose challenges to the effectiveness of such screening programmes.

Here we describe a model (called the COHVIN model) for combined hearing and vision (eye) screening of newborns in immunization clinics, which are attended by both hospital- and community-born babies, as a potential solution. 

## Methods

### Study design

The nonprofit organization Shomea developed the combined hearing and eye screening model. After obtaining administrative approvals, it conducted free newborn combined hearing and eye screening in two major hospitals between November 2021 and February 2024; demographic and clinical data were collected via structured questionnaires. We analysed data from Shomea’s screening retrospectively.

### Study setting

Cameroon, a lower-middle-income country in sub-Saharan Africa, has a scarcity and uneven distribution of resources needed to assess congenital hearing impairment,^18^ resulting in a lack of universal newborn hearing screening. One study on 582 children younger than 15 years of age found that 437 (75.1%) of the children had hearing loss,^19^ which was probably prelingual. 

The prevalence of retinoblastoma in a tertiary hospital in Cameroon was estimated at 0.83 per 10 000 consultations,^20^ in line with the reported rate in low-income countries of 0.84 per 10 000.^21^ Late diagnosis and a lack of tertiary level eye-care services able to conduct eye surgery for infants in Cameroon^22^ means that most cases of retinoblastoma are managed through enucleation (i.e. surgical removal of the eye).

#### Study sites

We chose immunization clinics offering the bacille de Calmette–Guerin vaccine, which has high uptake in sub-Saharan Africa (about 87%).^23^ As vaccinations are conducted twice a week, a single screening team with one set of equipment can cover two clinics on alternate days. This approach not only reduces costs but also ensures better handling of screening equipment compared to that by maternity personnel in busy hospitals.

We selected two immunization clinics based on closest proximity to Shomea’s regional office. To gain a better understanding of the challenges and opportunities associated with implementing the combined screening model within different categories of health facilities, one clinic was located at Bonassama district hospital, an important entry point for newborns within the primary health-care system; and the other clinic at Laquintinie hospital, a major referral hospital. Both hospitals are in Douala, the economic capital of Cameroon. These clinics serve a predominantly urban population of 10 000–30 000 people. In addition to the bacille de Calmette–Guerin vaccine, other vaccines, such as diphtheria–pertussis–tetanus and measles–rubella vaccines, are administered free of charge. According to Cameroon’s Expanded Programme on Immunization schedule, children receive between two and four different vaccines per visit and require five visits between birth and 11 months of age to complete their immunization.

### Description of the model

Shomea developed a combined screening model using the double diamond framework of divergent and convergent thinking,^24^ and tested it in immunization clinics before implementation. This model addresses human resource shortages, and bridges the gap between early screening and definitive care for newborns with hearing and/or vision impairments. Integrating these screenings into existing child health-care services, streamlines neonatal screening,^25^^,^^26^ and leverages the current high bacille de Calmette–Guerin vaccination coverage (> 90%) in Cameroon.^27^

A single team of trained auxiliary staff conducted combined hearing and eye screenings at immunization clinics. Such clinics are attended by hospital- and community-born babies. The staff also conducted screenings in neonatology units for high-risk newborns, with eligibility starting from two days after birth.

The trained auxiliary staff were unemployed graduates awaiting health-care jobs. To prepare them for this role, they underwent a two-week training programme covering basic anatomy and physiology of the ear and eye, the working principles of the screening instruments, and practical sessions to ensure proficiency in screening techniques. Training sets were small (5–15 screeners) and emphasized hands-on experience. Trainees could therefore practise on patients until they were comfortable at conducting the screenings independently.

### Screening setting

Screenings were conducted in a quiet, dark room specifically designed for sensory testing in babies, as recommended by the American National Standards Institute.^28^ Clinics designated a screening room away from other patients, with staff ensuring minimal noise. For eye testing, the room was darkened with curtains (to maximize pupil dilation), and the direct ophthalmoscope was fully charged. Cooperation from immunization clinic staff is crucial for maintaining these conditions. The estimated set-up cost per clinic was around 500 United States dollars (US$).

### Screening procedure

Screeners conducted screenings before vaccination to ensure that the baby was calm and to reduce its distress and crying, thus improving accuracy. The screening protocol used the otoacoustic emissions test for hearing, a simple, efficient indicator of cochlear function recommended by WHO for newborn screening.^13^ The fundal reflex test (also called the red reflex) was used to screen the baby’s eye; this can help diagnose cataracts, glaucoma, retinoblastoma and retinopathy of prematurity.^29^ The hearing screening was done first, as ideally babies needed to be asleep or in a quiet state; babies’ eyes needed to be open when screening their vision. The screening process took about 15 minutes in total.

#### Otoacoustic emission screening

Otoacoustic emissions testing uses a small probe inserted into the ear canal to transmit sound to the middle and inner ear. Cochlear outer hair cells generate a response detected by the probe’s microphone. Otoacoustic emissions tests assess cochlear function at 500–6000 Hz, with a sensitivity of 85–100%, specificity of 91–95%,^30^ a positive predictive value of 67% and a negative predictive value of 99%.^26^ In our study the screening unit provided an automated pass or refer result.

#### Fundal reflex screening

The fundal reflex test uses a direct ophthalmoscope. In our study the screener sat half a metre from the baby; they then shone a coaxial light through the baby’s pupils, rotating left and right to check the fundal reflex and detect other abnormalities. The screeners then shone a light over both eyes from one metre away to check reflex symmetry. Parents held infants in a cradle hold to keep them calm and stabilize their heads (i.e. the baby was positioned across the parent’s lap with their head facing upwards and resting on the holder’s forearm; the parent’s hand supported the length of the baby’s body). A toy or light behind the screener helped focus the baby’s gaze. A normal test showed symmetrical bright reflexes, while a reduced or absent reflex indicated obstruction.^31^

### Patient flow

We offered newborns that did not pass the first otoacoustic emissions test a free retest within three months. If they did not pass again, we referred them for an auditory brainstem response test. Newborns that did not pass the fundal reflex test were referred the same day to an ophthalmologist for a paid consultation covered by the patient’s family.

### Quality control

Regardless of their results, we sent every tenth child screened for verification and retesting by on-site specialists (ophthalmologists, optometrists, audiologists and ear, nose and throat surgeons).

### Ethical consideration

The Ethics Committee of the University of Douala approved this retrospective analysis. We conducted sensitization (i.e. telling the parents about the importance of hearing and eye screening) in two parts: first, in sessions of about 15 minutes for parents as a group, and later, individually, in the screening rooms (for about 5 minutes). Every parent provided written informed consent before screening; they were told that refusal to participate would not affect the quality of care received, and that the screening posed no risk to their babies.

### Study outcomes

Our study outcomes were: (i) the proportion of newborns that were referred for hearing and eye pathologies after the screening test; and (ii) the sensitivity, specificity and predictive values of hearing and eye screening conducted by trained auxiliary staff compared with specialists.

### Sample size estimation

Based on a 14.3% referral rate from a previous Nigerian study,^6^ and using a 95% confidence level with a 5% margin of error,^32^ we calculated the minimum required sample size as 168 newborns.

### Statistical analysis

We conducted statistical analysis using SPSS Statistics v25.0 (IBM, Armonk, United States) and calculated frequency counts, percentages, sensitivity, specificity and predictive values.

## Results

A total of 1807 newborns were screened at a median age of 13 days (interquartile range: 7–28 days). No parent declined screening. Table 1 summarizes the clinical characteristics of the study participants. Of them, 976 (54%) were female; 1717 (95%) were full-term deliveries; 54 (3%) preterm (i.e. 28–37 weeks); and 36 (2%) extremely preterm (< 28 weeks). Most of the babies (1789; 99%) were born in hospital, while 18 (1%) were delivered in the community by midwives or traditional birth attendants. We found no association between gender and failure in hearing or eye screening.

**Table 1:** Clinical characteristics of babies participating in a combined hearing and eye screening at vaccination clinics, Cameroon, November 2021 to February 2024

Parameter, variable	No. (%) (*n* = 1807)
**Type of delivery**	
Normal vaginal delivery	1256 (69)
Prolonged-labour vaginal delivery	23 (1)
Forceps-assisted vaginal delivery	9 (1)
Caesarean section	519 (29)
**Admitted to the neonatal intensive care unit**	
Yes	45 (3)
No	1762 (97)
**Prolonged neonatal jaundice requiring hospital admission**	
Yes	275 (15)
No	1532 (85)
**Family history of hearing impairment among siblings**	
Yes	56 (3)
No	1702 (94)
Don’t know	49 (3)
**Family history of visual impairment among siblings**	
Yes	181 (10)
No	1595 (88)
Don’t know	31 (2)

### Hearing test

One hundred and forty-one babies (8%) did not pass the otoacoustic emissions test in either one or both ears and were scheduled for a repeat test within three months. Only 39/141 (28%) of babies returned, of whom 13 (33%) did not pass the second-line test and were scheduled for an auditory brainstem response test.

Of the babies who did not return, 24 parents opted to seek additional assessments from specialist physicians (otorhinolaryngologists). The remaining 78 babies were lost to follow-up: 18 (23%) were unreachable, 32 (41%) relocated and 28 (36%) declined further testing, often citing lack of spousal support (especially from the father of the baby) due to social stigma associated with loss of hearing.

### Eye test

Among the 1807 newborns screened, 27 (2%) had an absent fundal reflex (bilateral in two cases), while 11 (1%) were uncertain. Rescreening of uncertain cases by optometrists confirmed that there were no instances of absent fundal reflex.

### Performance

To assess the performance of trained screeners, 150 children were rescreened by specialists to determine the sensitivity, specificity and predictive values of the screeners (Table 2 and Table 3). We classed values > 80% as high and values of 60–80% as moderate.

**Table 2:** Frequency distribution of hearing and eye screening by trained auxiliary staff versus specialists, Cameroon, November 2021 to February 2024

Trained screeners	Specialists		
	Refer	Pass	Total
**Hearing screening, no. of screens**			
Refer	9	1	10
Pass	2	138	140
Total	11	139	150
**Eye screening, no. of screens**			
Refer	6	1	7
Pass	3	140	143
Total	9	141	150

**Table 3:** Sensitivity, specificity, positive and negative predictive values of hearing and eye screening by trained auxiliary staff versus specialists Cameroon, November 2021 to February 2024

Measure	No./total (%)	
	Hearing screening	Eye screening
Sensitivity	9/11 (82)	6/9 (67)
Specificity	138/139 (99)	140/141 (99)
Positive predictive value	9/10 (90)	6/7 (86)
Negative predictive value	138/140 (99)	140/143 (98)

Screeners demonstrated high sensitivity (82%), specificity (99%) and predictive values (90% for positive and 99% for negative) for hearing screening; while they demonstrated moderate sensitivity (67%) and high specificity (99%) and predictive values (86% for positive and 98% for negative) for eye screening.

## Discussion

In this study we evaluated a combined screening model for newborns attending immunization clinics using trained auxiliary staff. The median age at screening was 13 days; this is well beyond the 48-hour period after birth that is associated with false positives.^33^ This age is also early enough to allow timely interventions (e.g. cataract surgery, which should take place within six to eight weeks of birth).^34^ We found that gender had no apparent impact on the study results or analyses.

Deliveries outside hospitals remain common in low- and middle-income countries. In a meta-analysis of Demographic and Health Surveys, seven countries in sub-Saharan Africa had proportions of home births greater than 50%.^35^ By conducting the combined screening at immunization clinics, we ensured that babies born in the community (such as at home) were included. A previous Nigerian study showed that mothers who deliver outside hospitals still attend immunization clinics.^6^ Community-born babies would have been missed in a purely hospital-based screening model, although in our urban study population, only 1% of infants were actually born outside the hospital. 

Trained auxiliary staff demonstrated high sensitivity, specificity and predictive values for hearing screening. Similarly, except for sensitivity, which we classed as moderately high, these indices were also high for eye screening. One explanation for the lower sensitivity of eye screening compared with screening of hearing is that good performance in fundal reflex screening requires practice. Even in high-income countries, midwives and nurses report unfamiliarity with the direct ophthalmoscope, especially in non-Caucasian babies.^36^^,^^37^ One study also described how the fundal reflex depends on light reflection from the posterior eye and its absorption by the retinal pigment epithelium, which varies in thickness across ethnicities, being thinner in Caucasian and Asian individuals than in individuals of African descent.^38^

In our study, a specialist detected an absent fundal reflex in three cases that were missed by trained auxiliary staff; two of these cases occurred early in the screening programme. This outcome underscores the need for an optimal setting for screening and well trained screeners who receive regular refresher training and monitoring. We postulate that screening by trained screeners may be more reliable than screening conducted by rotating maternity nurses, as the latter also have other duties. This approach may also lead to better handling of screening equipment and better equipment security.

The reported prevalence of absent fundal reflex can reach 22%, depending on the study population.^39^^–^^42^ In our study, we found the prevalence of an absent fundal reflex to be 2%.

Although infants who did not pass the initial otoacoustic emissions test were all invited for a retest, less than a third returned. On-site second-line auditory brainstem response testing could have reduced this loss as it would have confirmed hearing impairment without the need for another visit. The lack of costly auditory brainstem response machines at screening sites remains a major barrier to universal hearing screening in low- and middle-income countries, as recalling or referring patients often leads to high loss to follow-up. Conducting first- and second-line hearing tests in the same visit would ease the burden on screeners, who often struggle to reach patients for retesting. A low referral uptake may also stem from limited community awareness, which underscores the need for stronger sensitization efforts as part of screening programmes.

Alarmingly, we found that one third of the babies who were retested still did not pass the hearing test, this highlights the prevalence of hearing impairments among newborns in Cameroon. All of these babies were referred for auditory brainstem response tests under sedation and remain in the follow-up programme.

As vaccine clinics run twice weekly, our combined screening model allows a single screening team to serve two nearby clinics on alternate days with a single set of instruments, optimizing instrument use and covering a larger population with fewer devices than other screening models. The fifth working day could be reserved for targeted screening in paediatric intensive care units, where hearing loss and retinopathy of prematurity are more common.^43^

Currently, only fundal reflex screening is conducted among high-risk infants. We are in the process of gathering resources to train our screeners to capture fundus images in this group. The details and outcomes of this screening approach in paediatric intensive care units will be evaluated in a separate study.

A major limitation of our study was the low sensitization, especially among fathers, which might explain refusals for retesting. To address this, Shomea now enhances screening awareness through brochures at antenatal clinics, media campaigns and discussions at screening sessions. Another limitation of our study is that we only rescreened a small proportion of patients (150/1807; 8%) to assess the trained screeners' sensitivity, specificity and predictive values. Evaluation of a bigger sample may give better accuracy. A final limitation is that we did not assess the cost–effectiveness of the intervention. Future studies should address this gap.

Our study presents an insight into the prevalence of hearing and visual impairments in Cameroon and outlines a novel approach to universal newborn hearing and eye screening. The combined screening conducted at immunization clinics enhances coverage while optimizing resources by using a single, well-trained screening team. Costs are relatively low: we estimate one set of screening equipment (comprising devices for testing otoacoustic emissions, the auditory brainstem response, and an ophthalmoscope for assessing the fundal reflex) costs approximately US$ 12 000 and can serve two nearby vaccination centres. The setup cost for each screening site is around US$ 500, and salaries for screening staff are approximately US$ 400 per month per person.

Scaling up this intervention will require government support: preventive measures such as vaccination and screening are essential components of basic health care, which the government has the responsibility to fulfil. We suggest that the government could recruit, train and equip dedicated screeners at the national level and deploy them to immunization clinics, similar to vaccination staff. To ensure sustainability, partnerships with public health programmes, international donors or national insurance schemes could help integrate screening into routine maternal and child health services.

In conclusion, the combined screening model offers a promising screening approach for low-resource countries facing low sensitization, stigmatization and high rates of delivery outside hospitals.
